# Study protocol - Indigenous Australian social networks and the impact on smoking policy and programs in Australia: protocol for a mixed-method prospective study

**DOI:** 10.1186/1471-2458-13-879

**Published:** 2013-09-24

**Authors:** Raglan Maddox, Rachel Davey, Tom Cochrane, Ray Lovett, Anke van der Sterren

**Affiliations:** 1 Centre for Research and Action in Public Health, University of Canberra, University Drive, Canberra, ACT 2606, Australia; 2 Australian Institute of Aboriginal and Torres Strait Islander Studies, Australian National University, GPO Box 553, Canberra, ACT 2601, Australia; 3 Centre for Excellence in Indigenous Tobacco Control, University of Melbourne, 207 Bouverie Street, Melbourne, VIC 3010, Australia

## Abstract

**Background:**

Tobacco use is the most preventable cause of morbidity and mortality in Australia. Comprehensive tobacco control has reduced smoking rates in Australia from approximately 34 per cent in 1980 to 15 per cent in 2010. However, 46 per cent of Aboriginal and Torres Strait Islander people (Indigenous Australians) smoke on a daily basis, more than double the rate of non-Indigenous Australians. The evidence of effective tobacco control strategies for Indigenous Australians is relatively scarce. The aim of this study is to (i) explore the influences of smoking in Indigenous Australian people and to (ii) help inform and evaluate a multi-component tobacco control strategy. The study aims to answer the following questions: - do individuals' social networks influence smoking behaviours; - is there an association between various social and cultural factors and being a smoker or non-smoker; and - does a multi-component tobacco control program impact positively on tobacco behaviours, attitudes and beliefs in Indigenous Australians.

**Methods and design:**

Our prospective study will use a mixed-method approach (qualitative and quantitative), including a pre- and post-test evaluation of a tobacco control initiative. The study will explore the social and cultural context underlying Indigenous Australian tobacco use and associated factors which influence smoking behaviour. Primary data will be collected via a panel survey, interviews and focus groups. Secondary data will include de-identified PBS items related to smoking and also data collected from the Quitlines call service. Network analysis will be used to assess whether social networks influence smoking behaviours. For the survey, baseline differences will be tested using chi^2^ statistics for the categorical and dichotomous variables and t-tests for the continuous variables, where appropriate. Grounded theory will be used to analyse the interviews and focus groups. Local Aboriginal community controlled organisations will partner in the study.

**Discussion:**

Our study will explore the key factors, including the influence of social networks, that impact on tobacco use and the extent to which smoking behaviours transcend networks within the Indigenous Australian community in the ACT. This will add to the evidence-base, identifying influential factors to tobacco use and the effectiveness and influence of a multi-component tobacco control strategy.

## Background

Tobacco use is the most preventable cause of morbidity and mortality within Australia [1]. While Australia is a world leader in comprehensive tobacco control, including the recent implementation of the world’s first plain packaging policy through the Tobacco Plain Packaging Act 2011, there is room for improvement, especially in certain sub-populations [1,2]. Tobacco control policies in Australia have resulted in reducing the smoking rates from approximately 34 per cent in 1980 to 15 per cent in 2010 [1,2]. However, this is not the case for all population groups with 46 per cent of Indigenous Australians smoking on a daily basis, more than double the rate of non-Indigenous Australians [1]. Indigenous Australians have a notable history with tobacco [3,4]. For example, tobacco provided an incentive for labour with many Indigenous Australians continuing to receive rations of tobacco from employers up to the 1960s [4-6]. The high rates of smoking among Indigenous Australians [7-10], is the single most significant contributor to premature deaths (one in five) among Indigenous Australian people. Tobacco smoking also contributes significantly to shorter life expectancy when compared with non-Indigenous Australians [11].

It is well established that there are a number of cultural and socio-environmental factors that influence mainstream tobacco use [12-19]. Evidence has indicated that peer associations can impact on behaviours, including the initiation and cessation of smoking predominantly among young people, and in relation to substance use [20-22]. Smoking can be a mechanism to maintain and strengthen kinship bonds and social relationships and to enhance a sense of belonging and social cohesiveness [23-25]. Research has investigated socially, culturally and politically appropriate approaches to behaviour change in relation to tobacco use [26-31] with social networks theorised to have a significant influence in the behaviour change processes [32-36]. However, our understanding of attitudes, behaviours and the way in which social networks influence and operate in relation to smoking behaviours in Indigenous Australian communities is very limited. Our study aims to investigate various social and cultural factors and their influence on smoking behaviours, attitudes and beliefs. Measures of smoking behaviour include smoking status and levels of tobacco consumption, while indicators of attitudes and beliefs include:

•how often a respondent thinks about ‘enjoying smoking’;

•if respondents’ perceive cigarette brands to be more prestigious or more harmful than other cigarette brands; or

•the perceived level of importance of a number of statements, such as ‘smoking may interfere with my performance’, ‘smoking may make me vulnerable and put me at risk for harm’ or ‘my culture does not allow smoking’.

The study will use a number of underpinning theories, including the theory of triadic influence, social network analysis, diffusion of innovations theory and homophily in order to triangulate evidence and add validity to our interpretation of this complex issue [37,38]. This will assist to develop our ability to design and implement optimal, culturally appropriate and effective tobacco control targeting Indigenous Australians [39,40].

### Research questions

The research will investigate the impact of tobacco control programs and policies among the Indigenous Australian population in the ACT region and ask the following research questions:

•do individuals' social networks influence smoking behaviours?

•is there an association between various social and cultural factors and being a smoker or non-smoker?

•do tobacco control programs in the Australian Capital Territory (ACT) impact on tobacco behaviours, attitudes and beliefs in the Indigenous population?

### Underpinning theories

In recent times there has been a strong commitment to address the high rates of smoking in the Indigenous Australian population through the Close the Gap campaign, the National Tobacco Strategy 2012–2018, the National Partnership Agreement on Closing the Gap in Indigenous Health Outcomes and the National Healthcare Agreement [41-47]. The National Healthcare Agreement has set the target of closing the life expectancy gap for Indigenous Australians within a generation (2030) and to halve the 2009 Indigenous smoking rate by 2018 [42]. The ACT Government made a further commitment to reduce smoking rates among Indigenous Australians through the development of the ACT Aboriginal and Torres Strait Islander Tobacco Control Strategy 2010–2014 [41].

The Strategy recognises that while there is evidence regarding the prevalence of smoking in the Indigenous Australian community, reports on the effectiveness of tobacco control initiatives for Indigenous Australian people and communities are scant [48]. Much of the work to date in Indigenous Australian tobacco control draws two central tenets:

1. tobacco control is best delivered in the community setting; and

2. to be effective participation must be based in the social, work or family environment [41].

Evidence indicates that the social network structure can influence health behaviour and that normative and other peer influences transmitted through network ties can shape risk behaviours [13,49]. A better understanding of the relationship between Indigenous Australian social networks and smoking is required [13]. Our research will utilise four associated underpinning theories in a prospective study of smoking behaviours of Indigenous Australian people [37,38,50].

### The theory of triadic influence

The theory of triadic influence describes three streams of influence in relation to tobacco use:

1. cultural/environmental influences - community characteristics, media influences, legislation and policy;

2. social-situational or normative influences - including parent and peer influences and their attitudes, use of tobacco and characteristics of relationships; and

3. individual, person or biological influences - genetic, biological, personality variables, gender, ethnicity and age [51,52].

**Figure 1:**
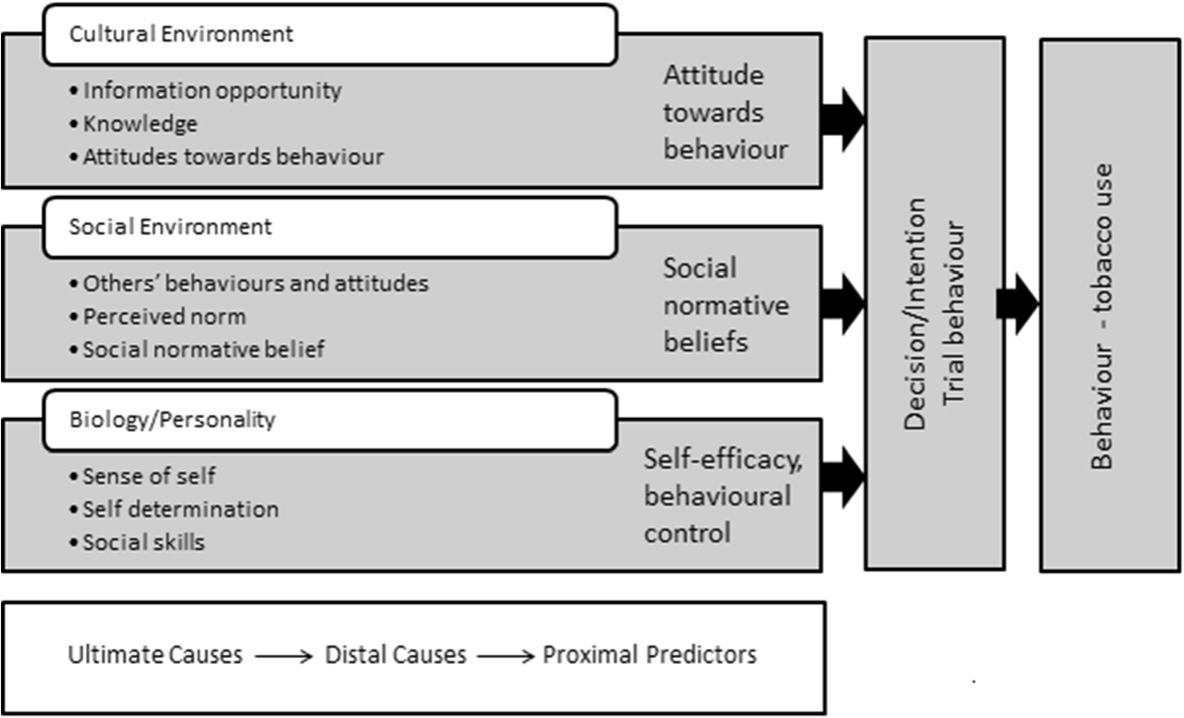
**The theory of triadic influence.** Source: Modified from [35,51].

**Figure 2:**
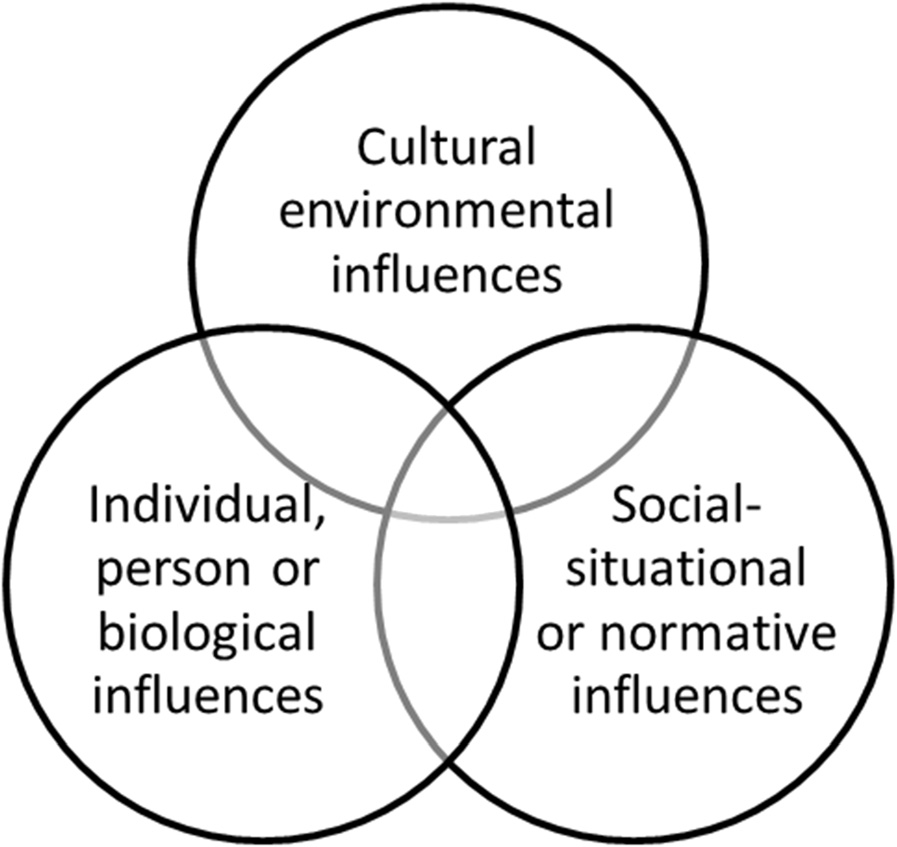
**The theory of triadic influence.** Source: Modified from [35,51].

As illustrated in Figures 1 and 2, the theory outlines three variables across the three streams of influence; ultimate, proximal and distal. Proximal factors influence behaviours directly (e.g. smoking related attitudes and beliefs) in contrast to ultimate factors, which are beyond the control of individuals, indirectly placing them at risk of smoking behaviour (e.g. broader cultural, social and biological influences) [51,52]. Given this context and based on research in relation to smoking among young people in the USA [20,21], it is expected that similar attitudes and beliefs will be reflected in participants’ broader social networks. Furthermore, it is this evidence and context that leads us to social network analysis, diffusion of innovations theory and homophily as directly and indirectly relevant theories regarding smoking among Indigenous Australian communities.

### Social network analysis

A network is a structure made up of nodes (individuals, organisations, etc) that are connected together by ties (relations such as friendship, kinship, exchanges, activities, etc) [53-55]. Social network analysis provides a way of characterising and investigating such structures, including through network visualisation (using graphic display as illustrated in Figure 2), structural analysis and statistical analysis [55-58]. If participants' networks influence smoking behaviours, it would be expected that participant’s networks would share similar smoking behaviours. Thus, we would compare the observed network to a simulated network with the same network characteristics, including the same overall rate of smoking prevalence, but with the incidence of smoking randomly distributed across the network [59]. If clustering is occurring among smokers or non-smokers, then the probability that a participant who smokes has a network contact who is also a smoker should be higher in the observed network than in the simulated network [21,60]. Perceived proportion of peers who smoke will be measured by analysing the respondents’ perceptions of how many of their peers smoke (about what proportion (%) of your friends and acquaintances use tobacco?) and respondents’ perceptions of how many of their five closest friends and family are regular smokers (thinking about your five closest friends and family, how many of these five are regular smokers?).

Wellman [61] outlined that individuals’ behaviour is best predicted by examining their social network and ecological characteristics in which they are entrenched; not intrinsic factors such as attitudes, drivers or demographic characteristics. Furthermore, nothing can be accurately understood in isolation or without context [61]. Therefore, these structures―social networks of interconnected individuals―and characteristics can be useful to develop, tailor and implement health promotion and public health programs, including tobacco control [62]. Within the Indigenous Australian context, tobacco was seen as a prestigious substance and has been a highly valued commodity [3,7,63]. Smoking has been a central mechanism for relationships, assisting to maintain and reinforce kinship bonds and social relationships and also used as a practical currency and an incentive prior to Indigenous Australian peoples full engagement with the cash economy in the late 1960s [4-6]. As a result, the primary data collection in this study is expected to reflect the importance of social and cultural norms regarding smoking and the influence of social networks. It is anticipated that similar behaviours and beliefs about smoking will resonate among participant networks [23-25].

### Diffusion of innovations theory

The diffusion of innovations theory is the most prominent behavioural application of network analysis and has been widely used in public health; explaining the steps, processes and how new ideas and practices spread within and between communities [64]. Given the context that has influenced the high rate of smoking among the Indigenous Australian community and the evidence regarding the social role of tobacco [3,4,7,63], it is logical that the diffusion of innovations theory will provide the theoretical foundations to investigate how social networks can affect behaviour and behaviour change around smoking [64]. Diffusion of behaviours and effective programs are a significant challenge for public health, health promotion and subsequently, tobacco use [65,66]. Ryan and Gross [67] identified significant influence on social contacts, interactions and interpersonal communication on the adoption of new behaviours. New behaviours and practices may originate in a community and can be disseminated and diffused through the community where they originated and beyond through numerous communication channels, such as mass media, social media, interpersonal channels and electronic communication [58,68,69]. As a result, it is evident that factors influencing diffusion are not static factors of behaviour change. Influential factors are generally dynamic interactions that occur between a number of factors, individuals and the environment [64]. If effective public health programs, products and practices are not effectively disseminated and diffused, they will not achieve optimal impact to improve public health [64].

### Homophily

Individuals' social networks can be homogeneous with regard to socio-demographic characteristics, intrapersonal factors or behaviours, such as smoking; “similarity induces homophily” [70]. Homophily is the principle that interaction between similar individuals or organisations occurs more frequently than among dissimilar individuals or organisations [70]. This is related to the process of peer socialisation, whereby people take on the values and behaviours of the ‘group’ in order to be accepted [71]. In the context of the social norms around tobacco among Indigenous Australian people and the high rate of smoking, homophily is a sound underpinning for this research. McPherson and colleagues [70] indicated that behavioural, cultural, genetic or other information that flows through networks is more likely to be clustered. In alignment with the theory of triadic influence, this may be one factor for the inconsistent effect of tobacco control on smoking rates of different population groups [70].

### Synthesis: underpinning theories

Our research will use triangulation to enhance the validity and generalisability of the study by increasing the likelihood that the findings and interpretations will be credible and dependable [37,38,50,72]. Triangulation will strengthen the research and lead to a more comprehensive understanding of the complex issue of tobacco use, using multiple disciplinary and theoretical lenses to view and investigate the research findings and data sets [37,50,73].

Triangulation was originally used in the health and social sciences by psychologists Campbell and Fiske [74] using multiple tests to measure the same constructs to look for convergent validity. It has been used in a broad range of research related to health sciences and within the public health and health promotion sphere [75-80]. Our research will use these underpinning theories, data sources and analyses to enhance the validity of the study [37,38,50,72]. There is limited evidence regarding effective tobacco control for Indigenous Australian people and insufficient evidence in relation to network analysis in this area. It is this gap in knowledge that motivates the research questions. Therefore, this research will strengthen our understanding of the factors that influence smoking, including exploring cultural and social beliefs and attitudes.

## Methods/design

### Aim and objectives

The objectives of the research are to answer the following questions:

•do individuals' social networks influence smoking behaviours;

•is there an association between various social and cultural factors and being a smoker or non-smoker; and

•do the tobacco control programs under the Action Area 1 of the ACT Aboriginal and Torres Strait Islander Tobacco Control Strategy 2010–2014 (this includes smoking cessation groups, youth and community health promotion programs and education campaigns) impact on tobacco behaviours, attitudes and beliefs in the Indigenous Australian population.

We will undertake surveys, focus groups and interviews in two waves approximately 12 months apart; pre- and post-implementation of the multi-component tobacco control initiative. This will assist to identify commonalities and disparities, assessing the effectiveness of program and further exploring socio-environmental mechanisms that influence tobacco use, attitudes and behaviours [81,82]. Data will be collected via:

1. surveys;

2. interviews;

3. focus groups; and

4. use of existing de-identified health data, for example, the Talking About the Smokes survey data, data regarding relevant Pharmaceutical Benefit Scheme (PBS) item listings related to smoking (e.g. item codes for nicotine replacements) and Quitlines call data and volume.

### Data collection instruments: survey, interview guide and focus group guide

The data collection instruments were developed based on valid, reliable and tested surveys, including the:

•Australian Census;

•Fagerström Test for Nicotine Dependence;

•National Aboriginal and Torres Strait Islander Social Survey questionnaire;

•National Aboriginal and Torres Strait Islander Health Survey questionnaire; and

•National Drug Strategy Household Survey questionnaire [2,83-88].

The network analysis components are adapted from previous studies of social networks by Alexander et al. [89], De Lange et al. [90] and others [14,90-94]. Each instrument includes social network questions in relation to the characteristics of the participants’ friends, family and people that reside in their household. The data collected, including responses to the network questions, will provide invaluable insight into the centrality of participants, their relationships/networks and smoking behaviours and beliefs. Network analysis will also be supported through recruitment via convenience and snowball sampling.

#### The sample population

Participants will generally be Indigenous Australian people residing in the ACT region. The sample will include adults and children (12 years and above). Young people have been included to reflect the younger Indigenous Australian demographic profile and the early uptake of tobacco use in children [95,96].

#### The sampling frame

Our primary points of recruitment in the ACT include the ACT Indigenous Network, an Aboriginal Community Controlled Health Organisation, an Aboriginal Community Controlled Youth Centre, local community events, and a number of other Indigenous Australian organisations and their networks. These organisations will be used to help recruit participants via convenience and snowball sampling [37,97]. After potential participants receive the study information sheet and voluntarily make contact with the researcher to participate, the potential participants will be asked to provide informed consent prior to participating in the study.

### The survey

The survey (paper and online versions) will collect quantitative data on individuals’ behaviours, attitudes and ecological characteristics, including social network data to explore the influence of family and peers. Therefore, while the participants are the source of all information, there are two different sampling units: the individual respondent; and the relationships/networks [98]. Objectives of the survey will include the domains; demographics; socio-economic status, and will explore:

•factors that influence smoking, including cultural and social beliefs and attitudes related to smoking, smoking cessation and non-smoking;

•attitudes, knowledge, beliefs and awareness in relation to smoking behaviours;

•nicotine dependence (Fagerström Test for Nicotine Dependence) [83,84];

•the impact of tobacco control programs and campaigns, including awareness and recognition;

•smoking, quitting and non-smoking behaviours; and

•family, friends and peers influence in relation to smoking behaviours.

#### Sample size

A minimum sample size of 102 participants was determined. This sample size is sufficient to obtain 90 per cent power to detect a 10 per cent reduction in smoking between the pre-intervention group (36%) when compared with the post-intervention group. The current population smoking rate is based on data obtained from the 2008 National Aboriginal and Torres Strait Islander Social Survey (NATSISS) relating to the ACT Indigenous Australian population [87].

#### Analysis

Statistical and social network analysis will be used to characterise and describe the results, using multiple imputation of missing data prior to analysis [55,81]. In examining the association between various social factors, data will be aggregated and entered in SPSS, UCINET, NetDraw and Microsoft Excel for statistical and network analysis. In assessing if tobacco control programs have influenced behaviours, attitudes and beliefs in relation to smoking, analysis will incorporate common descriptive statistics and comparisons between the pre- and post-intervention groups. For example, comparisons between the pre and post-intervention groups will use X^2^ (categorical distributions) and T-tests (interval or ratio data). Bivariate associations between variables will be tested by X^2^ in analysing smoking type (daily smoker, occasional smoker, light smoker, social smoker, ex-smoker and non-smoker) by gender, age group, income group, education level, etc. Wilcoxon rank sum tests or Spearmans rank correlations will also be used depending on whether the variables are binary or ordinal. Multiple regression will be used to test whether individual variables are independently predictive of outcomes. Comparisons will be conducted across and between the sub-groups for both pre- and post-intervention [81,99]. Analysis will also include examining the data from the Fagerström Test for Nicotine Dependence questions in the survey for reductions in means scores, indicating reduced nicotine dependence. The higher the accumulated Fagerström score per participant, the more intense the participant’s physical dependence on nicotine [84]. Computations will be undertaken using SPSS and Microsoft Excel [83-85].

Social network analysis will be used to assess if individuals' social networks influence smoking behaviours. Network analysis will include exploring smoking and non-smoking networks constructed from the survey data and complemented by the qualitative data collection. This will include network visualization, structural analysis and statistical analysis [55]. Several network level measures of structure will be assessed, including clustering, network size, number of ties and reciprocity [55,58]. To study the clustering of smoking behaviour, we will compare the observed network at each data collection point to a simulated network with the same network characteristics, including the same overall rate of smoking prevalence, but with the incidence of smoking randomly distributed across the network [59]. If clustering is occurring among smokers or non-smokers, the probability that a participant who smokes has contact with other smokers should be higher in the observed network than in the simulated network [21,60]. These network metrics will be used to provide a descriptive presentation of the network/s and any changes over time. The pre- and post-test survey will be analysed using Analysis of Variance (ANOVA) on the gain scores and Analysis of Covariance (ANCOVA).

### Interviews and focus groups

The interview component of the research study aims to collect in-depth qualitative data on individuals’ behaviours, attitudes and ecological characteristics, including exploring potentially more sensitive factors, such as the influence of family and peers. It is expected that the interviews will expand on the depth of survey findings, broadening the perspective of contextual factors and their influence on tobacco use [50,81]. The use of open ended questions will provide broader scope and more detailed and enriched qualitative data on the determinants of tobacco use, including both barriers and enablers to tobacco use [50,82]. As outlined in the *Synthesis: underpinning theories*, analysis of a range of data sources, data collection methods and the weight of evidence is expected to provide a more comprehensive view of tobacco use [50]. Thus, each form of data collection―survey; interviews; focus groups; and existing data collections―will independently provide part of the story for the research aim, objectives and research questions, but together, they will contribute to a higher level of analysis and a more comprehensive understanding of tobacco use of Indigenous Australians living in the ACT [50,81].

Objectives of the interview component include investigating and determining knowledge of the existence and content of the multi-component tobacco control strategy, and investigating and exploring in particular:

•smoking, quitting and non-smoking behaviours; and

•is there an association between various social determinants, such as education and employment, and being a smoker or non-smoker. This will include exploring social norms and the influence of social networks.

#### Sample size

The sample size of the interview component (and the focus group component) of this study is based on theoretical saturation. Thus, an exact sample size for the project can only be ascertained as the project progresses, as it will be based on the numbers required for the data to be rich and detailed enough to support thematic analysis. Theoretical saturation will be achieved through collecting data across a diverse range of participants to fully flesh out ideas and themes, until no new themes emerge [37,81,97,100]. Theoretical saturation also indicates the development of categories in relation to their properties and other characteristics, including variation [37,81]. It is anticipated that at a minimum, a sample of 25 participants will be required based on previous studies, such as the "Starting to Smoke" Experiences of Indigenous Youth study [101]. We will offer multiple days, times and locations to participate in the interviews, working with the community organisations and potential participants to ensure participation is as convenient as possible.

#### Analysis

In investigating the influence of social networks on smoking behaviours; the impact of tobacco control programs in the ACT on tobacco behaviours; and if there is an association between some social factors and being a smoker or non-smoker, we will follow some procedures and principles of grounded theory [100,102]. This is to ensure that we do not shift concepts into incongruent situations. Grounded theory involves grounding text in the context that it was constructed [100,102]. Grounded theory will form the underpinning conceptual framework that informs the analysis for the interview and focus group components of this research. The grounded theory approach will be modified as the research project is primarily descriptive in outcome, rather than theory generating. Grounded theory utilises a systematic, inductive research process to generate grounded theory that emerges through constant comparative analysis of qualitative data [37,103]. This "general method of comparative analysis" results in systemic theory, identifying core variables that are grounded in the collated and synthesised data; assisting to interpret the data [104], [105]. Glaser [104] explained that “grounded theory has the purpose of generating concepts and their relationships that explain, account for, and interpret the variation in behaviour (sic) in substantive area under study” [19,104]. Four fundamental criteria formed the basis for the methodology: fit, modifiability, relevance and work. *Fit (valid)*―grounded theory emerges from the analysis of data gathered from the system; therefore, the theory fits and is relevant. *Modifiability (control)*―grounded theory is induced from the interviews and associated documentation, thus, the theory closely reflects what is actually happening and is highly applicable [37,100,103]. *Relevance (understanding)*―as grounded theory fits and is relevant, it is readily understandable to the people interacting with the field because it portrays the latent patterns within the field [37,100,103]. *Work (generality)*―grounded theory fits the field, is relevant, and is understood by people within the field, it is important to understand that grounded theory produces theory, not description [37,100,103].

The research project is primarily descriptive in outcome, rather than theory generating and therefore, selected parts of the grounded theory process will be utilised during the research project. This modified grounded theory approach will include data collection through interviews and focus groups, which will be transcribed for coding, analysis and compilation of the findings [100]. Coding will include constant comparison, documentation and identification of themes throughout the findings, including core categories and sub-categories. The selective coding will also include constant comparison and documentation resulting in dense, saturated core categories. The core categories will be sorted, documented and described [37].

**Figure 3:**
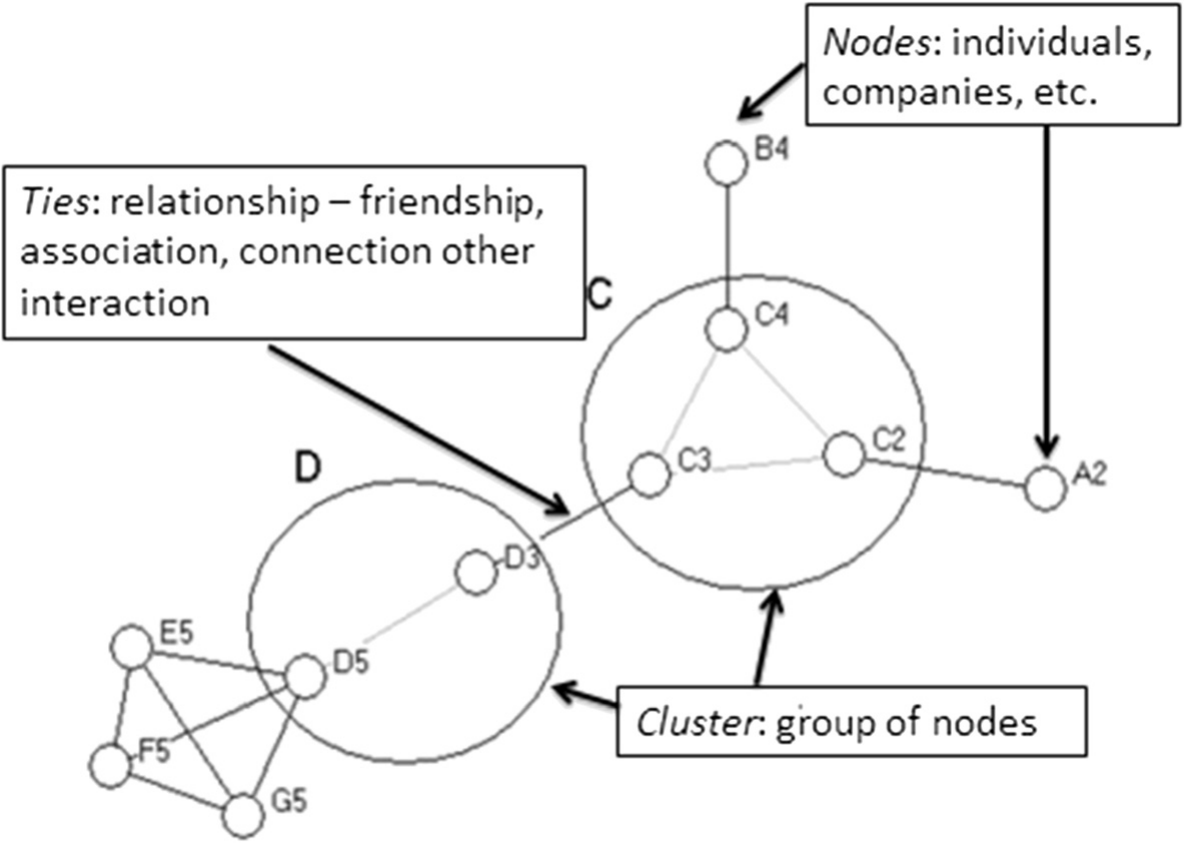
Network infrastructure.

**Figure 4:**
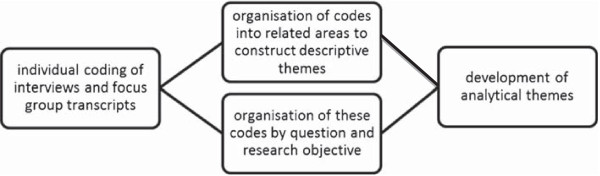
Analysis process of interviews and focus groups.

The interviews will follow an interview guide―informed by components of the National Aboriginal and Torres Strait Islander Social Survey, Health Survey and the National Drug Strategy Household Survey to address the research aim and objectives―to ensure methodological consistency. The interviews will be transcribed verbatim from electronic recordings. The transcripts will be coded using QSR Nvivo 10 and crosschecked with field notes. QSR Nvivo 10 will be utilised in coding each sentence according to meaning and content, supporting the thematic synthesis. As outlined in Figure 3, the text and codes will contribute to capturing the meaning and content of the interviews and each sentence. This will assist to identify similarities and differences, as abstract and analytical themes emerge, grouping the codes in a rational structure. The interview guide and the research objectives will also be utilised to group the sentences to ensure comprehensive analysis. This cyclical process will be repeated until no new themes emerge; adequately describing and explaining the aim and objectives of the research [37,103-106]. As outlined in Figure 4, the use of sentence coding will also assist to synthesize the qualitative research and recognise the concepts from individual interviews [37].

### Focus groups

In complementing and expanding the other methods of data collection, the focus group component aims to generate more of a “real world” group dynamic with peers and to gain a combined local perspective from multiple viewpoints. This will help to explore the influence of social networks on smoking beliefs and behaviours and investigate if there is an association between some social factors and being a smoker or non-smoker. The use of focus groups can help generate new thinking and allow for a broader perspective of contextual factors in relation to the influences of tobacco use. As outlined, our research will utilise a number of data sources and analyses to enhance the validity of the prospective study, increasing the likelihood that the findings and interpretations will be credible and dependable [37,38,50,72]. Triangulation will strengthen our research and lead to a more comprehensive understanding of the complexity of tobacco use and tobacco control [37,50,91,107]. Thus, the objectives of the focus group component of the research run parallel to the other components of the research, further investigating and exploring in particular the influence of peers and social norms in relation to smoking behaviours.

#### Sample size

As with the interview component, the sample size of the focus group component of this study is based on theoretical saturation as previously outlined [37,97,100]. The focus group interviews will include a small group of participants, approximately 6 to 12 people of similar age, similar smoking habits or as deemed socially and culturally appropriate. For example: smokers, ex-smokers, non-smokers, men’s groups, ‘mums n bubs’ and youth groups. The focus group interviews will be approximately an hour in length and held at convenient locations for the participants, such as the participant’s office, clinic, the university or a nearby location.

#### Analysis

The analysis of the focus groups will align with the interview analysis, informed by the procedures and principles used in grounded theory to ensure that concepts are not shifted into incongruent situations [100,102]. The focus groups will follow a focus group guide to ensure methodological consistency. In investigating if social networks influence smoking beliefs and behaviours, if ACT tobacco control programs impact on tobacco behaviour and if there is an association between some social factors and being a smoker or non-smoker, we will follow some procedures and principles of grounded theory. Each focus group session will be transcribed verbatim from electronic recordings, with the transcripts coded using QSR Nvivo 10 and crosschecked with field notes. The text and codes will contribute to capturing the meaning and content of the focus groups, assisting to identify similarities and differences, as abstract and analytical themes emerge; grouping the codes in a logical structure. The focus group guide and the research objectives will also be utilised to group sentences and themes [37,103-106].

### Existing data collections

A number of existing data collections will also be used in our study. These data collections will assist in triangulation, complementarity and integration of the quantitative and qualitative data [50,78,82,108]. The pre-existing data collections are expected to include de-identified data collected from health organisations, including:

•Talking About the Smokes data―Talking About the Smokes is a national survey modelled on the International Tobaccciso Control Policy Evaluation Project to improve the understanding of smoking and quitting behaviours within the Indigenous Australian community [109]. The Project has been adapted to suit the context of smoking cessation and tobacco control for Indigenous Australians, and includes a data collection site within the ACT region [109];

•PBS items―the PBS is part of the Australian National Medicines Policy and provides affordable access to necessary medicines for Australians [110]. There are a number of nicotine replacement therapy (NRT) items listed on the PBS (NRT items – 3414Q, 5571 F, 5572G, 5573H) to assist smokers with nicotine withdrawals and to help smokers make a quit attempt [110]. The volume of NRT items accessed through the PBS can be monitored by jurisdiction, including within the ACT [110]; and

•Quitline call data―Quitline is a telephone services that aims to offer treatment and provide timely information that will help smokers make a quit attempt [111]. De-identified data could include call volume, call volume by post code and number of quit attempts.

#### Analysis

Analysis will incorporate descriptive statistics and comparisons between the pre- and post-intervention groups, including usage patterns of relevant PBS items and Quitline call volume. For example, post-intervention comparisons between the pre and post-intervention groups using chi-square and T-tests will be carried out. It would be expected that there would be increased uptake of NRT on the PBS post intervention and increased calls to Quitlines [81,99]. Analysis will include assessment of the means, ranges and rates to identify commonalities and disparities between this existing data collection and the primary data collected through the survey, interviews and focus groups. The available data from Talking About the Smokes will influence what sort of analysis can be undertaken. This will be explored in due course. Computations will be undertaken using SPSS and Microsoft Excel for statistical analysis [83-85].

### Ethical review

The project has been informed by and is in compliance with the World Medical Association Declaration of Helsinki, the National Statement on Ethical Conduct in Human Research, Values and Ethics - Guidelines for Ethical Conduct in Aboriginal and Torres Strait Islander Health Research and Guidelines for Ethical Research in Australian Indigenous Studies [112-114]. The project takes into account the sensitivities around sampling Indigenous Australian people aged 12 years of age and older. A key ethical component and integral facet of the study is community engagement. In engaging with the community, we are also partnering with Winnunga Nimmityjah Aboriginal Health Service, a community controlled health organisation and working with other community stakeholders in the area. The research received ethics approval from the University of Canberra Human Research Ethics Committee (Project number 12163) and the ACT Health Human Research Ethics Committee (ETH10.12.232).

## Discussion

There is a challenge ahead if we are to achieve the ambitious ‘Close the Gap’ Campaign for Indigenous Health Equality target, to close the health and life expectancy gaps between Indigenous Australians and non-Indigenous Australians within a generation (2031) [47,115] and to halve the 2009 smoking rate of Indigenous Australian people by 2018 [42]. It is expected that this research will have benefits for the Indigenous Australian health sector and the community in terms of adding to the evidence for what might influence smoking behaviour and to help inform future tobacco control interventions.

Our understanding of attitudes, behaviours and effective tobacco control and the influence of how social networks influence smoking in Indigenous Australian communities is very limited. Social networks are theorised to significantly influence behaviour change processes. Through this project, we expect to contribute new knowledge about factors influencing tobacco use among the Indigenous Australian community.

### Limitations

Whilst the ideal study design would be one that included a randomised ‘control’ group it is not practical or possible due to resource constraints to run such a study when the intervention is aimed at all Indigenous Australian people living in the ACT. In order to address some of these limitations, we propose using a mixed-methods approach that offers a range of perspectives on a program's processes and outcomes and a greater understanding of the findings.

Pre-test and post-test design are not as robust, but they are widely used and accepted in behavioural research for the purpose of comparing groups and/or measuring change resulting from experimental treatments or interventions.

## Conclusions

The importance of people’s social context in relation to smoking and our understanding of the influence of social networks for health behaviour change process in the Indigenous Australian population is very limited. This study aims to fill a gap and add to the evidence to help close the health and life expectancy gaps between Indigenous Australians and non-Indigenous Australians [47,115]. The research will increase our understanding of:

•individuals' social networks and their impact on beliefs, attitudes and behaviours in regards to smoking;

•any association between various social factors and being a smoker or non-smoker; and

•the effectiveness of tobacco control programs under the ACT Aboriginal and Torres Strait Islander Tobacco Control Strategy on tobacco behaviours, attitudes and beliefs.

Given the recent commitment to address the high rates of smoking in the Indigenous Australian population through the Close the Gap campaign, the National Tobacco Strategy 2012–2018, the National Partnership Agreement on Closing the Gap in Indigenous Health Outcomes, the National Healthcare Agreement and the ACT Aboriginal and Torres Strait Islander Tobacco Control Strategy 2010–2014 [41], the results of this research could be of interest to a number of stakeholders. These include policy makers, General Practitioners and other health professionals such as Regional Tobacco Coordinators, Tobacco Action Workers, Quitlines, Aboriginal Health Workers, General Practitioners and other Allied health professionals who are engaged with addressing smoking, or should be engaged with addressing smoking, through Indigenous programs and policy initiatives. Furthermore, the results will potentially inform the design of tobacco control programs and policies and may influence the sector’s ability to meet the Close the Gap targets and the National Healthcare Agreement goal to halve the 2009 smoking rate of Indigenous Australian people by 2018 [42,47].

## Competing interests

The authors declare that they have no competing interests.

## Authors’ contributions

RM^1^ is a PhD Candidate who conceived the study and participated in the design involved in drafting and finalising the manuscript. RD^1^ participated in the design of the study, drafting the manuscript and revising it critically for important intellectual providing final approval of the version to be published. TC1 contributed to the design of the study, with particular input on analysis and interpretation of data. TC has been involved in drafting the manuscript and revising it critically for important intellectual content. RL^2^ is a Wongaibon man and has been involved in the preliminary discussion around the acquisition of data, contributing in the design of the study and will be involved in the analysis and interpretation of data. RL was also involved in drafting the manuscript and revising it critically for important intellectual content. AVDS^3^ contributed in the design of the study and was involved in drafting the manuscript and revising it critically for important intellectual content. All authors read and approved the final manuscript.

## Pre-publication history

The pre-publication history for this paper can be accessed here:

http://www.biomedcentral.com/1471-2458/13/879/prepub
